# Temporal Dynamics of Reactive CO_2_ Flow in Carbonate Rock: Insights from 4D Synchrotron Imaging

**DOI:** 10.1021/acs.energyfuels.5c02297

**Published:** 2025-10-09

**Authors:** Azibayam J. Amabogha, Amin Taghavinejad, Waleed Dokhon, Shashidhara Marathe, Lin Ma, Branko Bijeljic, Martin Blunt, Muhammad Arif, Yihuai Zhang

**Affiliations:** † James Watt School of Engineering, 3526 University of Glasgow, Glasgow G12 8QQ, United Kingdom; ‡ Department of Earth Science and Engineering, 4615 Imperial College London, London SW7 2BP, United Kingdom; § 120796 Diamond Light Source Ltd, Harwell Campus, Didcot OX11 0DE, United Kingdom; ∥ Department of Chemical Engineering, The University of Manchester, Manchester M13 9PL, United Kingdom; ⊥ Department of Petroleum Engineering, 105955 Khalifa University, Abu Dhabi 127788, United Arab Emirates

## Abstract

This research examines the dynamics of reactive CO_2_ transport in carbonate rock, focusing on the impact of carbonic acid-induced formation damage. We provide real-time visualization of these processes by employing four-dimensional (4D) high-resolution synchrotron imaging at the I13 beamline hosted at the Diamond Light Source. We visualize and quantify the temporal effects of reactive CO_2_ transport at the pore scale in carbonate rock. The experiment involved injecting CO_2_-saturated brine through the sample with in situ scanning to track the different stages of chemical dissolution. Analysis of the images shows a channelled dissolution pattern which corresponds with a gradual increase in porosity due to pore structure changes. Pore network models were generated from the segmented images to carry out a sequence of drainage and imbibition simulations. The result demonstrated that reduced capillary entry pressure with increased pore connectivity after dissolution. Furthermore, the trapping efficiency was quantified to predict a slight decrease in dissolution as the pores become broader and better connected.

## Graphical abstract

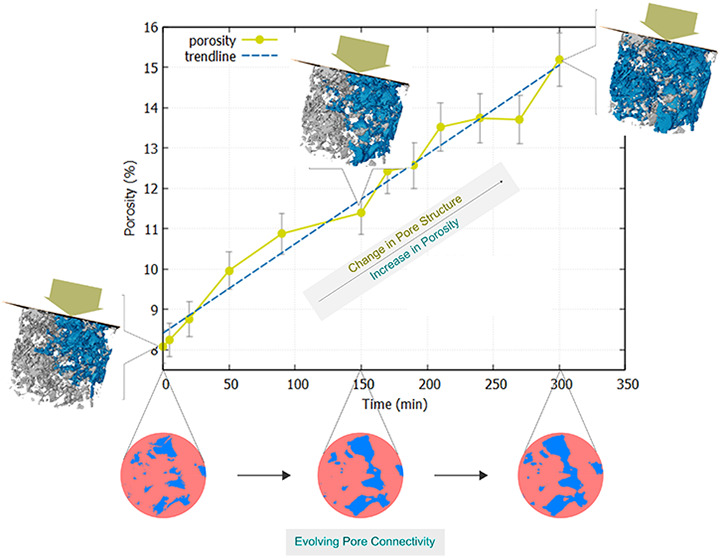

## 1 Introduction

Geologic carbon dioxide storage involves trapping CO_2_ from greenhouse emissions in underground geological formations for long-term sequestration.^1,2^ The depleted oil and gas reservoirs are targeted stored formations due to the existing infrastructure can largely reduce the costs.^3–5^ Additionally, given that these reservoirs have stored hydrocarbons for millions of years, it is reasonable to assume that they can similarly retain CO_2_ indefinitely. Carbonate reservoirs are good candidates because they contain a significant portion (60%) of the world’s oil reserves and provide ample storage space in sedimentary basins.^6^

However, injecting CO_2_ into carbonate reservoirs encompasses several key challenges. For example, most of these rocks contain formation water, which can interact with the injected CO_2_ to produce acidic brine (i.e., CO_2_-saturated brine) that reacts with carbonate minerals and can affect formation integrity, injectivity, and consequently the efficiency of CO_2_ storage.^7–9^ This process, referred to as reactive transport, involves complex interactions within the CO_2_-water-rock system. The ionic composition in the formation water is often complex, and when CO_2_ interacts with rocks, it can alter the composition, posing challenges for predicting multiphase flow.^10^ CO_2_ dissolves and chemically reacts with certain minerals in the rock, changing the rock’s pore-throat configuration and presenting a heterogeneous chemical reaction challenge.^9,11^ These reactions, involving dissolution and precipitation, can modify the rock’s pore structure and, thus, its porosity and permeability, as well as its overall capillary trapping capacity.^12^

Several studies have investigated the reactive transport process in carbonate rocks; however, most of these studies have been performed under static conditions. For example, Lebedev et al.^13^ used a lab-based micro-CT imaging technique to reveal changes in pore structures due to dissolution of calcite. Under actual CO_2_ sequestration conditions, the process is far from static: the injection of CO_2_ induces evolving multiphase flow, and the associated reactive transport processes are time dependent. For example, as CO_2_ dissolves in formation water to produce acidic brine, the continuous flow causes ionic composition and pH variations, affecting the reaction kinetics with carbonate minerals. Studies by Menke et al.,^14^ Voltolini and Ajo-Franklin^8^ and Yang et al.^15^ have highlighted the significance of these reactions under dynamic conditions. Their study confirmed that the dissolution patterns evolve nonuniformly due to the interplay between advection, reaction kinetics, and pore structure changes. Menke et al.^14^ demonstrated that localized dissolution can lead to the formation of preferential flow paths (channelling), significantly altering permeability over time. Similarly, Yang et al.^15^ confirmed that permeability evolution is highly dependent on flow pathways: samples with a single dominant flow path exhibited a higher power law exponent, meaning even slight changes in porosity cause dramatic shifts in permeability. In contrast, more homogeneous and fractured samples showed permeability changes that were more closely linked to variations in connected porosity. Additionally, Voltolini and Ajo-Franklin^8^ observed that evolving capillary pressure variations influence the migration and trapping of nonwetting phases, emphasizing the dynamic nature of CO_2_ sequestration. Field observations also strongly support the need for dynamic experimentation. For example, according to time-lapse three-dimensional (3D) seismic surveys conducted at the Sleipner CO_2_ storage site, plume evolution depends sensitively on subtle permeability contrasts and dissolution-driven connectivity rather than injection rate.^16^ At the same time, pilot projects at Wallula (USA) have shown that local dissolution and pressure evolution can alter migration pathways over time.^17^

Similar coupling of geochemistry and flow has also been observed in other storage contexts. Sennaoui et al.^18^ examined carbonate-rich and silicate-rich cores from the Bakken Formation and showed that mineralogy strongly controls CO_2_–brine–rock interactions: calcite-rich samples buffered acidity through calcite dissolution, while silicate-rich samples experienced clay dissolution, quartz precipitation, and ultimately salt crystallization in macropores that reduced brine mobility and pore connectivity. Fatah et al.^19^ combined carbonate core-flooding with molecular dynamics simulations and demonstrated that gas type plays a key role in controlling displacement behavior. CO_2_ injection produced the highest water recovery and differential pressure, while H_2_ produced the lowest, reflecting differences in viscosity and interfacial tension. Their simulations confirmed that all gases behaved as nonwetting phases, with variations in capillarity governed by interfacial tension and pore structure. Al-Yaseri et al.^20^ used X-ray-monitored core-flooding in sandstones. They found that, although average gas saturations ranged from 25 to 40%, residual gas saturations were effectively zero for all tested gases. In these high-permeability rocks, wettability and capillary forces were not only the dominant controls; instead, structural trapping governed storage behavior. Collectively, these studies emphasize that heterogeneity, mineral variability, and pore structure exert first-order control on dissolution pathways, permeability/porosity evolution and trapping efficiency across different lithologies. As a result, these coupled physicochemical activities can profoundly alter reservoir properties and storage security.^16^ However, a comprehensive understanding of these processes under reservoir-relevant dynamic flow conditions is still lacking.

One critical factor influencing these coupled processes is the flow rate used in the studies, which was higher than typical reservoir conditions and more akin to conditions expected near the wellbore. Under typical reservoir conditions, flow rates are of 10 orders of magnitude lower, meaning that diffusive transport and reaction kinetics may play a more dominant role compared to advection. The lower flow rates in deeper formations could result in more homogeneous dissolution than the strong heterogeneous channelling observed in laboratory studies. Moreover, the impact of capillary forces in controlling CO_2_ migration and trapping could be more pronounced under low-velocity flow conditions, affecting the overall efficiency of structural and residual trapping mechanisms.

This study addresses these gaps by conducting 4D high-resolution synchrotron imaging under controlled dynamic flow conditions that better represent pore-scale reservoir conditions. Furthermore, the images are then integrated into a pore-network model, allowing us to simulate evolving pore structures rather than assuming static geometries directly. This combined imaging–simulation approach provides a more accurate prediction of multiphase flow properties, including capillary pressure, relative permeability, and CO_2_ migration dynamics, because it accounts for the time-dependent changes in connectivity and pore-throat geometry that govern storage performance.

## 2 Materials and Methods

### 2.1 Rock and Fluid Properties

The carbonate rock Indiana limestone was used in this study because it is a widely studied analogue in reactive transport research, owing to its well-characterized mineralogy, predominant calcite composition, and high reactivity to CO_2_-acidified brine.^8,21,22^ And the carbonate rocks also account for a significant portion of the world’s hydrocarbon reservoirs, the abandoned carbonate oil and gas reservoir offer a great potential in carbon Geosequestration^19,23–25^

The Indiana limestone mainly comprises calcite-cemented grain stones of fossil fragments and oolites. In this study, samples with a diameter of 5 mm and a length of approximately 20 mm were used. Published X-ray diffraction data on the Indiana limestone describe it as a heterogeneous rock comprising 88 wt % calcite, 11 wt % magnesium, and 1 wt % quartz.^21^ A 3% wt. KI brine was used in the experiment. This brine was subsequently equilibrated with CO_2_ under high-pressure conditions (8 MPa pore pressure, 50 °C) using the HTHP reactor to produce the live brine. The pressure and temperature conditions were selected to simulate those expected at typical subsurface reservoirs, similar to those found at CO_2_ storage sites like Sleipner and Weyburn.^26,27^ Under these conditions, CO_2_ remains in a supercritical phase, allowing significant interactions with formation brines and carbonate minerals.^28^ The pH of the CO_2_-saturated brine after equilibration was measured using a portable pH meter to be approximately 3.6, consistent with values reported in other studies.^29–31^

### 2.2 Experimental Apparatus

#### 2.2.1 HPHT In Situ Core Flooding

A high-pressure and high-temperature core flooding system was combined with synchrotron imaging to investigate the CO_2_-brine flow dynamics and rock-fluid interactions within the Indiana Limestone. The schematic of the High Pressure-High temperature (HPHT) in situ core flooding apparatus is presented in Figure 1: the main components including high-pressure pumps, a core holder, a CO_2_ cylinder, a temperature control system, and a reactor. Four Teledyne Isco pumps were used to control the confining pressure, back pressure, as well as to inject brine. The limestone sample was placed in a PEEK micro core holder, which maintained pressure using confining fluids (water). The sample itself was encased in a Viton sleeve and connected to PEEK tubing through two steel end pieces.

**Figure 1:**
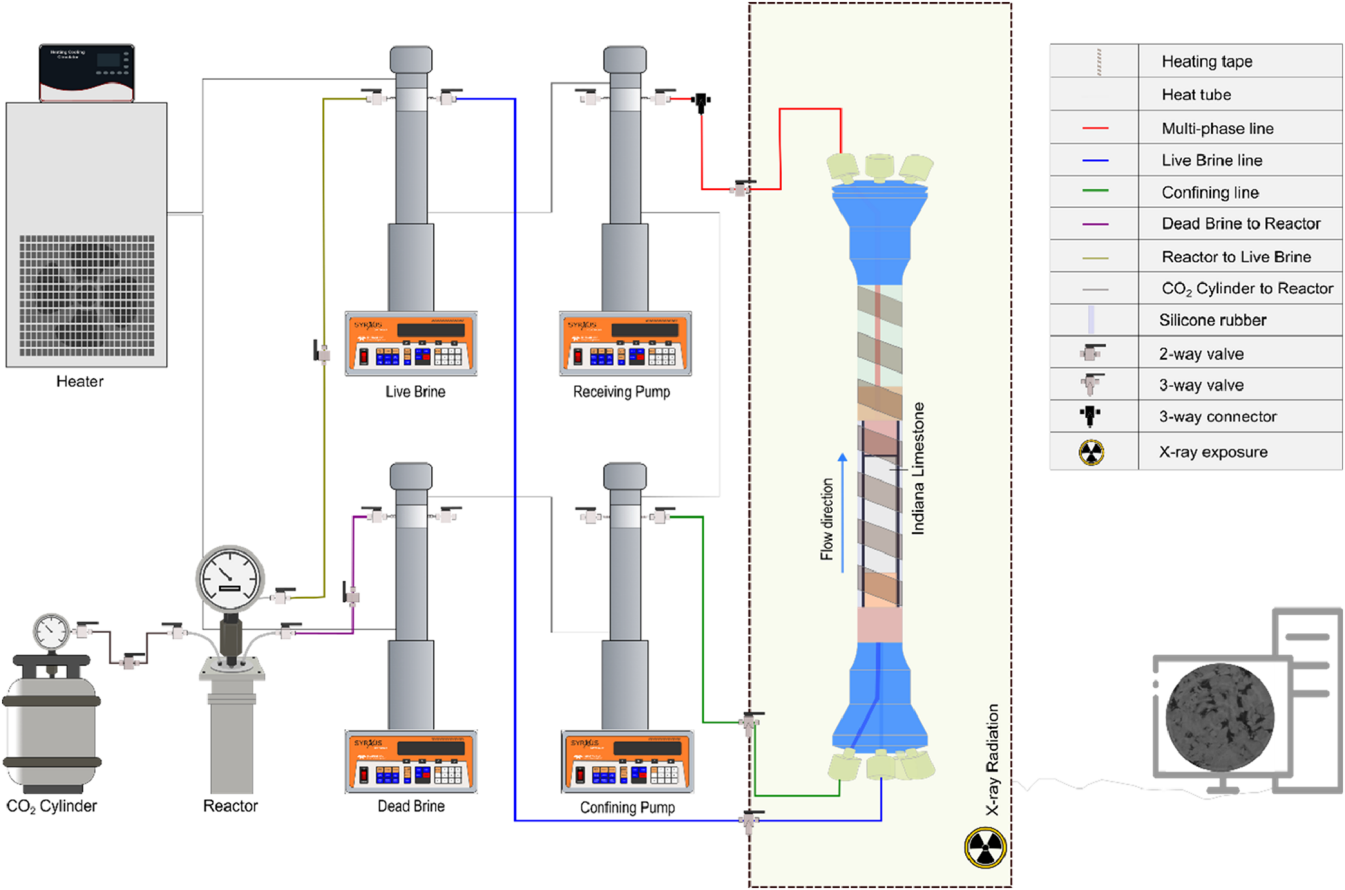
State-of-the-art high pressure-high temperature (HPHT) in situ X-ray core flooding apparatus.

#### 2.2.2 Synchrotron Computed Tomography

Synchrotron-based X-ray computed tomography (CT) was employed to obtain high-resolution 3D images of the rock and fluid phases during the experiment. The imaging and experiment were carried out at the I13 beamline at Diamond Light Source, Oxfordshire U.K., which operates within an energy range of 8–30 keV. The scans were captured using a 2× objective lens with a 4.2 mm × 3.5 mm field of view, and an exposure time of 0.3 s, producing 2,510 projections over a 180° rotation. This arrangement provided an effective pixel size of 1.625 μm. A 180° rotation was used instead of 360° due to concerns that the high-pressure tubes connected to the core holder could become tangled during a full rotation.

### 2.3 Experimental Procedure

The flow experiment was performed following the steps below.

1. The core and tubing system were vacuumed for 24 h to ensure that no air was left inside the system.
2. All pumps were continuously heated to 50 °C (323 K) using heating tubes that circulate warm water. The core holder was heated using electric heating tapes to the sample temperature.
3. The dry core was initially imaged at a voxel size of 1.625 μm under a confining pressure of 10 MPa. This was to enable the capture of a high-quality unsaturated image for both pore-space analysis and comparison with images capturing saturation and the progression of reactive transport effects.
4. Live brine was prepared in a reactor by vigorously mixing dead brine with supercritical CO_2_ for 1 h at 8 MPa and 50 °C. The resulting solution was then stored in one of the ISCO pumps for use.
5. Subsequently, the core was full saturated with dead brine with back (pore) pressure 8 MPa, then injected with the live brine solution at a flow rate of 0.04 mL/min.
6. 12 X-ray tomographs were captured over a 5-h period to monitor the dynamic reactive transport process in real time. A summary of the imaging specifications is given in Table 1 below.

**Table 1:** Experimental Conditions

rock type	experimental time	# of scans	# of projections	exposure time (seconds)	flow rate (mL/min)	time per scan (min)	resolution (microns)
Indiana limestone	300 min	12	2510	0.3	0.04	3	1.625

### 2.4 Image Processing and Analysis

Following scanning, the images were processed with image analysis tool, Avizo, to improve image clarity and extract quantitative data, as shown in Figure 2. The scans were carefully aligned, then they underwent denoising using a 3D nonlocal means filter, as proposed by Buades et al.,^32^ to enhance image clarity.

**Figure 2:**
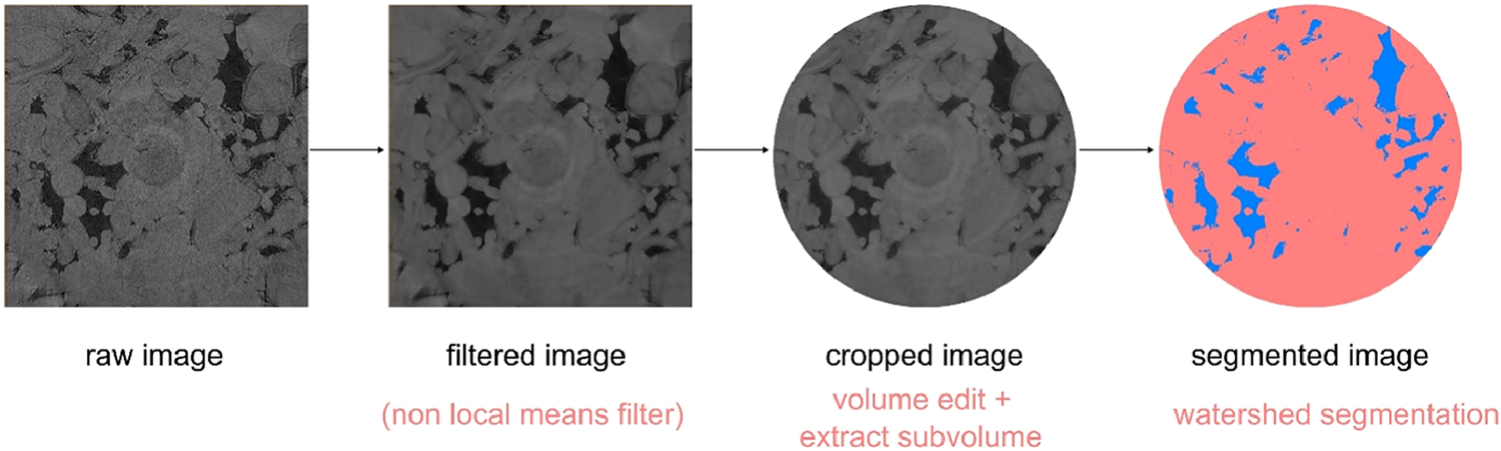
Workflow for image processing.

Subsequently, a watershed algorithm, as described by Schlüter et al.,^33^ was applied for phase segmentation, following the methodology outlined by Golab et al.^34^ This enables a separation of the pore area from the rock grain as shown in Figure 2. It is important to note that the rock grain of the Indiana limestone comprises of multiple mineral phases; however, these were all segmented as solid. The segmented images were subsequently inputted into a network extraction code^35,36^ to make model predictions of the change in permeability.

## 3 Results and Discussion

### 3.1 Pore Structure Evolution

Upon initial inspection of the unsegmented X-ray images, noticeable alterations in the rock’s microstructure can be observed. The entire sequence of the process is presented in Figure 3, with each image representing a step in the total experiment time of 5 h. Dissolution processes became evident as CO_2_-saturated brine flowed through the limestone, resulting in increases in pore size and connectivity. The comparison of the dry and postinjection scans shows the removal of material from the pore walls, due to CO_2_-brine-rock interactions. These observations are more visible in the segmented image and the 3D volume render presented in Figures 4 and 5 respectively, where an apparent expansion of the pores can be observed corresponding with increases in the volume of connected pores­(Figure 6).

**Figure 3:**
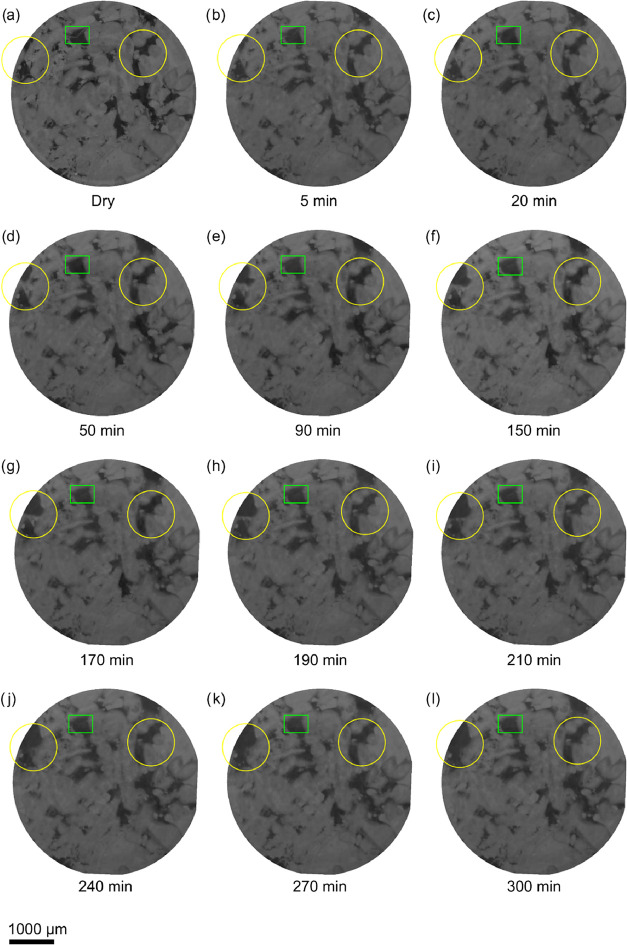
Two-dimensional cross sections of three-dimensional images. The yellow sections highlight examples of regions where dissolution has occurred. The green squares show where a small fragment exhibits preferential dissolution compared to other more solid blocks.

**Figure 4:**
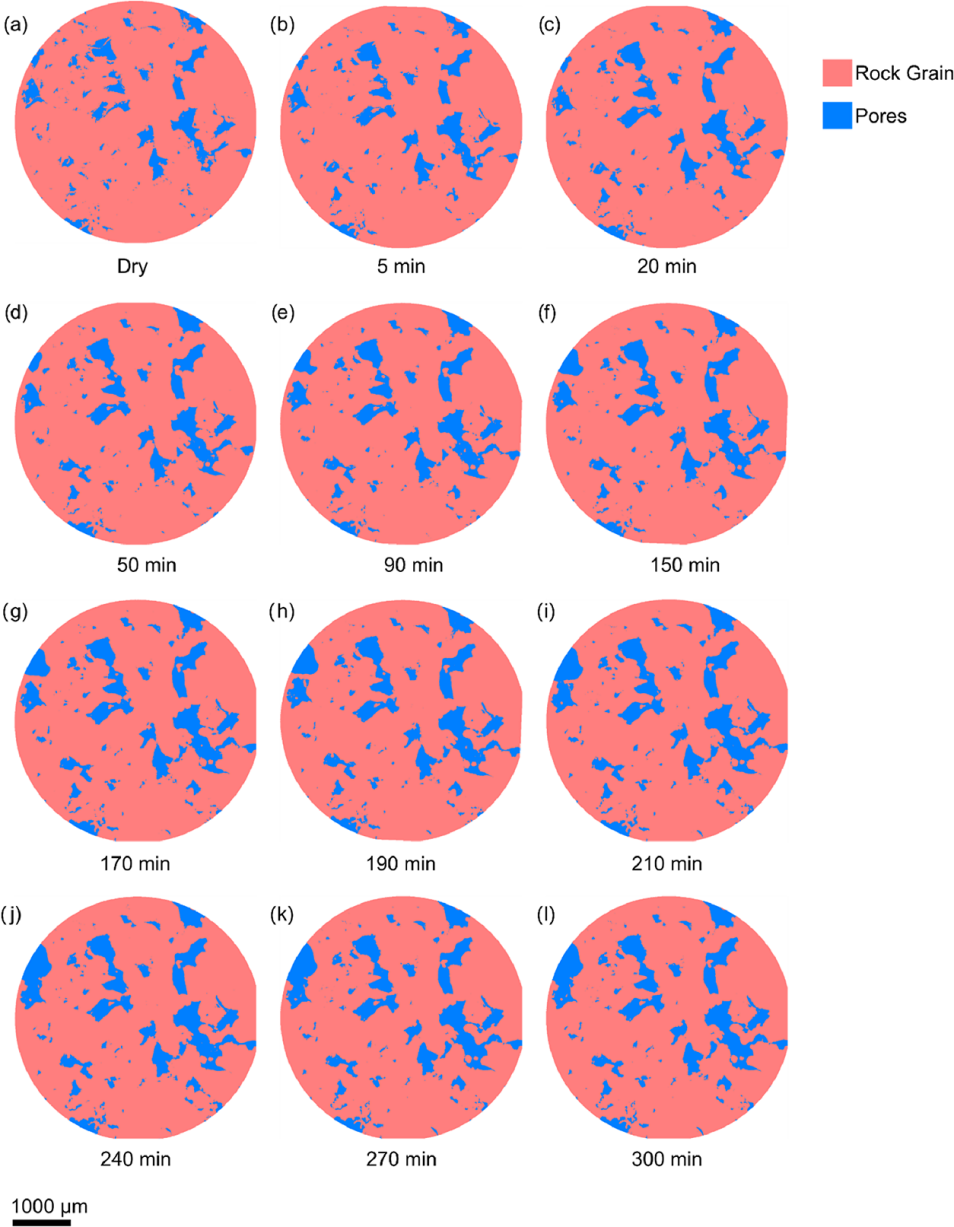
Two-dimensional evolution of pore structure over time due to mineral dissolution visualized through segmentation. The series (a–l) shows the changes in pore geometry (blue) and rock grain matrix (red) at different time intervals during the reactive transport process.

**Figure 5:**
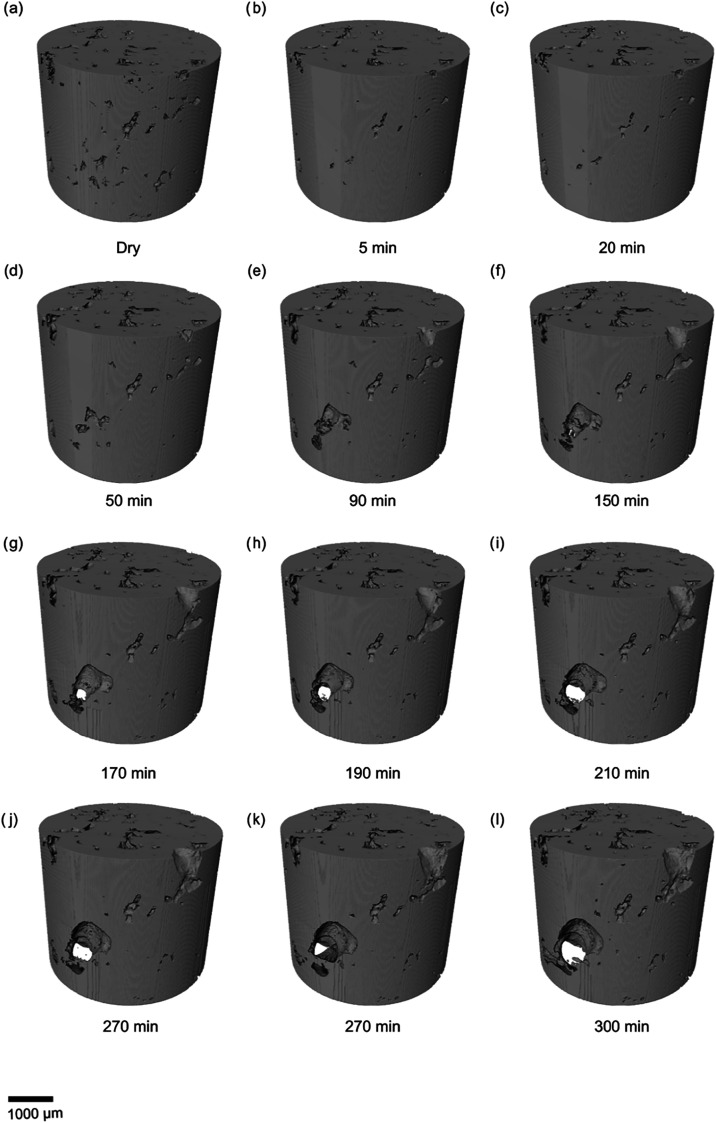
Three-dimensional volume render of rock structure showing expanding pores due to dissolution.

**Figure 6:**
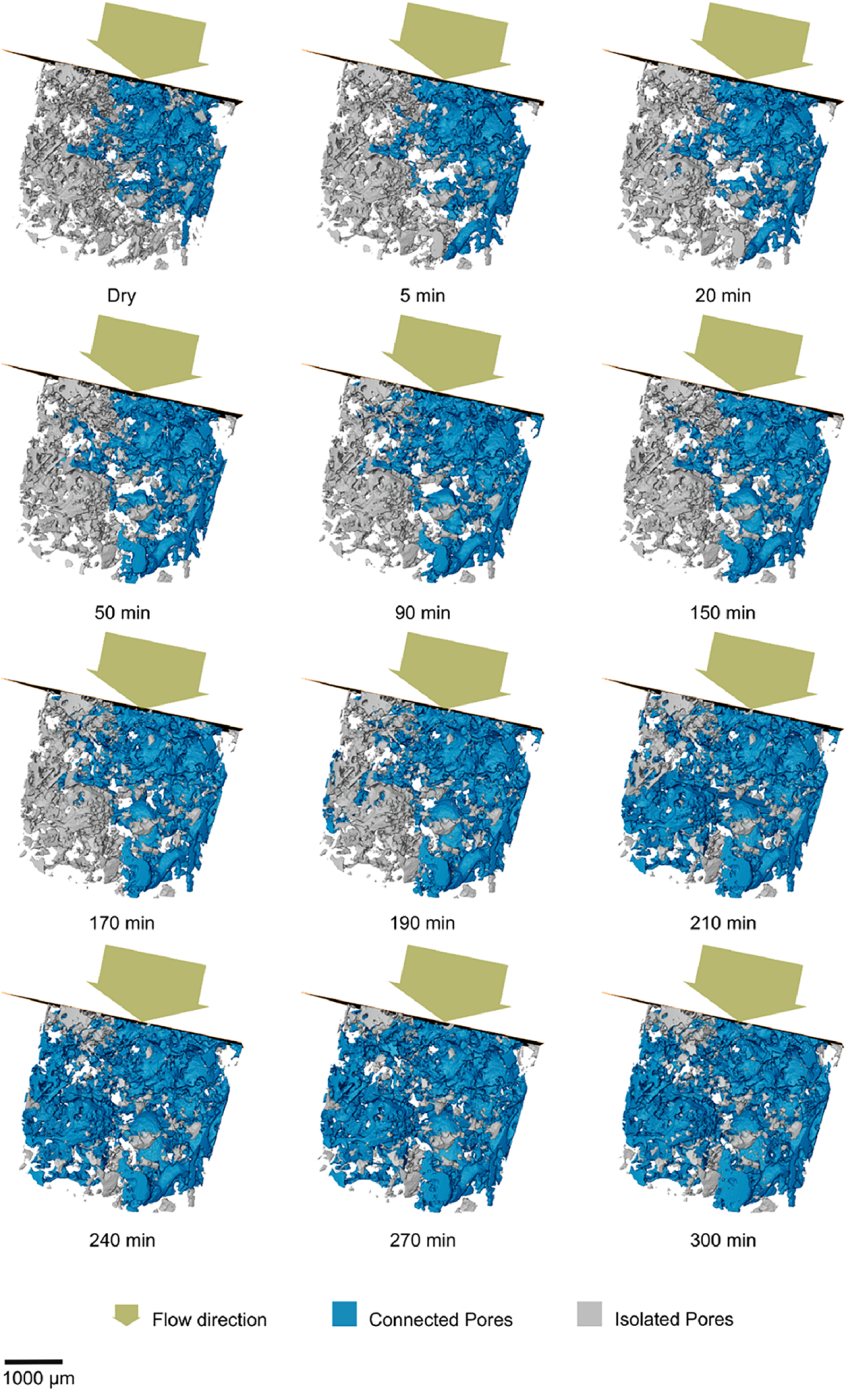
Pore space evolution over time shows connected and isolated pores.

The images show a preferential dissolution pattern, where thin fragments exhibit quicker dissolution rates than more solid structures. This observation aligns with findings from a similar study by Voltolini and Ajo-Franklin,^8^ which accurately attributed these regions to having a higher reactive surface area relative to their volume, making them more prone to dissolution. Similarly, some pores appeared to expand more rapidly than others, which suggests that the flow of carbonic acid through the sample was not uniform. This is likely due to preferential flow paths, with connected pores allowing for quicker fluid movement, leading to higher concentrations of CO_2_-rich brine in those areas than others.

### 3.2 Porosity Change

The porosity of the sample was estimated by computing the volume fraction of the pore space. Figure 7 shows the average porosity changes over the reactive transport period. Initially, the resolved porosity of the sample was measured at 8.08% and increased to 15.19% by the end of the experiment. The increase was approximately linear in time, indicating a broadly constant reaction rate. This type of behavior corresponds with the channelling effect observed in similar experiments by Menke et al.^7^ and Voltolini and Ajo-Franklin.^8^ It is important to note that since porosity was estimated solely from segmented synchrotron CT images, it reflects only the pore space that is above the imaging resolution (1.625 μm); as such, smaller micropores and nanopores, particularly those within fine-grained or intergranular regions, were not captured in this analysis. This means the reported porosity values likely underestimate the total porosity of the sample. Additionally, any reactive surface area associated with these subresolution features would not be accounted for, which could potentially affect the accuracy of derived quantities, such as permeability evolution.

**Figure 7:**
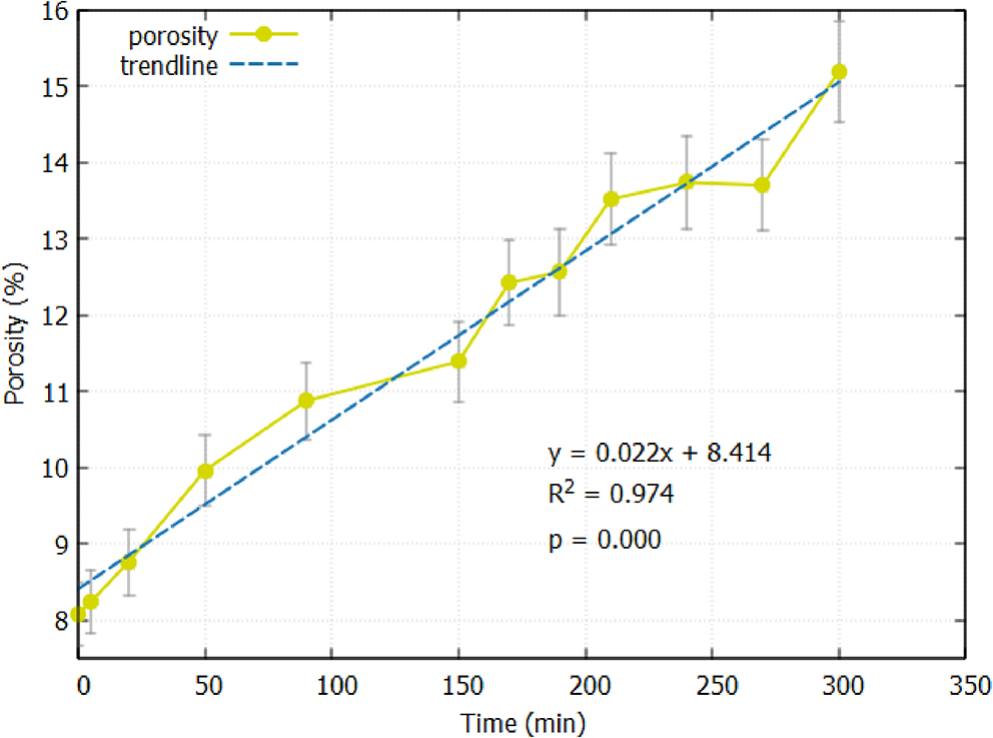
Average resolved porosity in the sample as a function of time.

### 3.3 Absolute Permeability

Pore network extraction was applied directly to the segmented images to analyze changes in geometry, topology and permeability resulting from the dissolution process. The model is based on the maximal ball algorithm which essentially identifies the largest possible spheres (or “maximal balls”) that can fit within the pore space.^35^ Previous studies have effectively validated the use of the maximal-ball algorithm in constructing network models.^37–40^ These spheres are used to represent the pores, while the connections between them define the throats. This method provides a topological mapping of the pore structure, enabling the characterization of flow paths and permeability alterations.

Permeability was then predicted using a network model simulation,^35^ and the results are presented in Figure 8. As expected, the permeability increases over time, but with a sublinear trend. This implies that permeability only increases gradually with an increase in porosity, which differs from previous work that observed a much steeper trend.^8,41^ This observation aligns closely with the notion that resolving a power law fit between porosity and permeability using a constant α value may not fully capture the range of pore structural changes that occurred during dissolution. That means that local morphological variations appear to affect how new pore space translates into enhanced flow paths, indicating that a single exponent may be inadequate.^42^ A more nuanced or multiexponent model could better account for the evolving changes in this case as measured in Bernabé, et al.^43^

**Figure 8:**
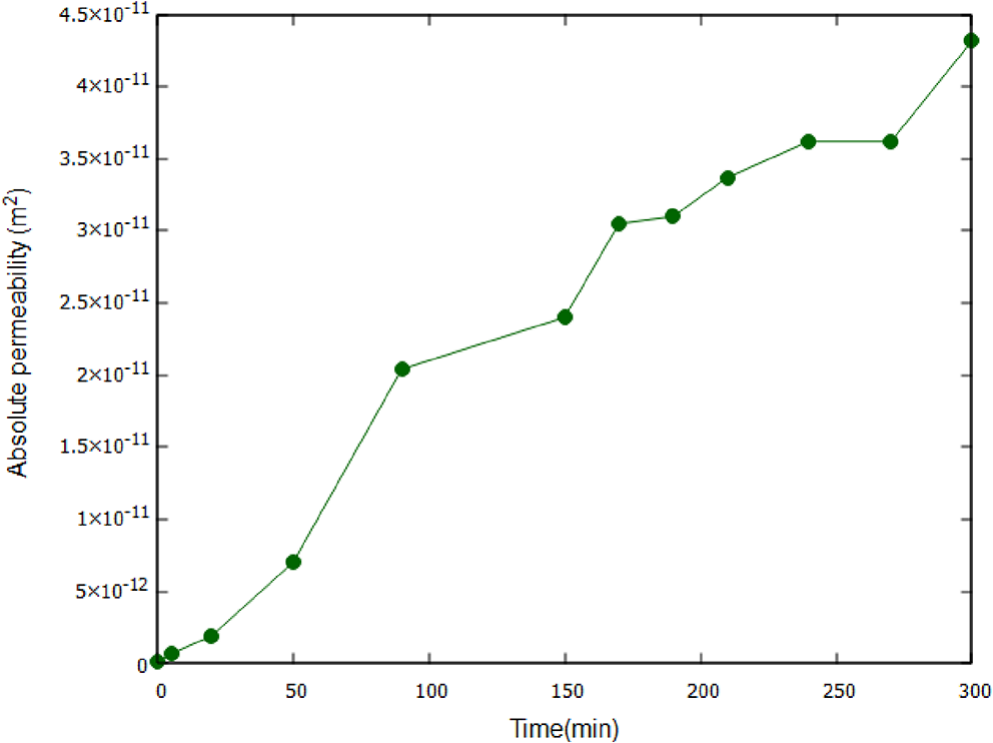
Computed permeability calculated on an extracted network model as a function of time.

### 3.4 Dimensionless Numbers and Reaction Rates

Damköhler (*Da*) and Péclet (*Pe*) numbers are two dimensionless values used to define the balance between reaction rate, fluid flow, and transport processes within a reactive system. *Da* estimates whether either reaction or transport is dominant by computing the ratio between reaction rate and advection. In this case, a low *Da* value (*Da* < 1) would indicate that calcite dissolution during the reactive process is slow relatively to the flow of CO_2_-rich brine. In contrast, when *Da* is high (*Da* > 1), it suggests that the reaction rate is fast compared to the flow, meaning that the dissolution of calcite occurs rapidly as CO_2_-rich brine moves through the porous medium. *Pe* on the other hand defines the ratio of advection and diffusion rates. A low Pe value (*Pe* ≪ 1) would indicate that the transport of CO_2_ brine is mediated by diffusion than advection.

*Da* and *Pe* was computed following the method described in Menke et al.,^41^ Menke et al.^14^ and Voltolini and Ajo-Franklin^8^

1 $$\mathrm{Da}=\frac{\pi r}{u_{\mathrm{av}}n}$$

*r* is the reaction rate for calcite which we take to be 8.1 × 10^–4^ mol·m^–2^·s^–1^ as measured in the experimental study by Peng et al.^44^ under similar conditions.

*n* is the number of moles of calcite per unit volume estimated using *n* = ρ_calcite_[1 – ϕ_total_]/*M*_calcite_ with ρ_calcite_ given as density of calcite (2.71 × 10^3^ kg·m^3^) and *M*_calcite_ as the molecular mass of calcite (0.1 kg mol^–1^).

*u*_av_ is the average interstitial velocity of the fluid. This is calculated from $u_{\mathrm{av}}=\frac{Q}{A\phi }$ ; where *Q* is the volumetric flow rate, *A* is the area perpendicular to the main direction of flow, and ϕ is the porosity estimated from the CT images.

*Pe* is defined as

2 $$\mathrm{Pe}=\frac{u_{\mathrm{av}}L}{D}$$

where *L* is the characteristic length defined as *L* = π/*S* following Mostaghimi et al.^45^ and *S* [m^–1^] is the specific surface area: the surface area divided by the bulk volume of the sample [m^3^]. The surface area was measured directly from the segmented images. *D* is the molecular diffusion coefficient of calcite which is measured at 25̊C (7.5 × 10^–10^ m^2^·s^–1^).^46^

From the calculations, *Da* was measured to range from 2.58 × 10^–3^ initially to 5.25 × 10^–3^ at the end of the experiment as shown in Figure 9a. Generally, the low *Da* values indicate that the reaction rate was considerably slower than advection. On the other hand, *Pe* was between 12.44 and 28.57 generally decreasing with time, indicating that advection was the dominant transport mechanism throughout the experiment. Throughout the experiment, the slight increases and decreases in *Da* and *Pe*, respectively, with time, reflect gradual shifts in the interplay between reaction and transport processes. As dissolution occurs, the local concentration of CO_2_-saturated brine evolves, and porosity changes, altering both the reaction kinetics and the velocity field. These subtle adjustments lead to a modest rise in *Da*—though it remains well below 1, indicating a comparatively slow reaction rate—and a reduction in *Pe*, yet advection continues to dominate the overall fluid movement.

**Figure 9:**
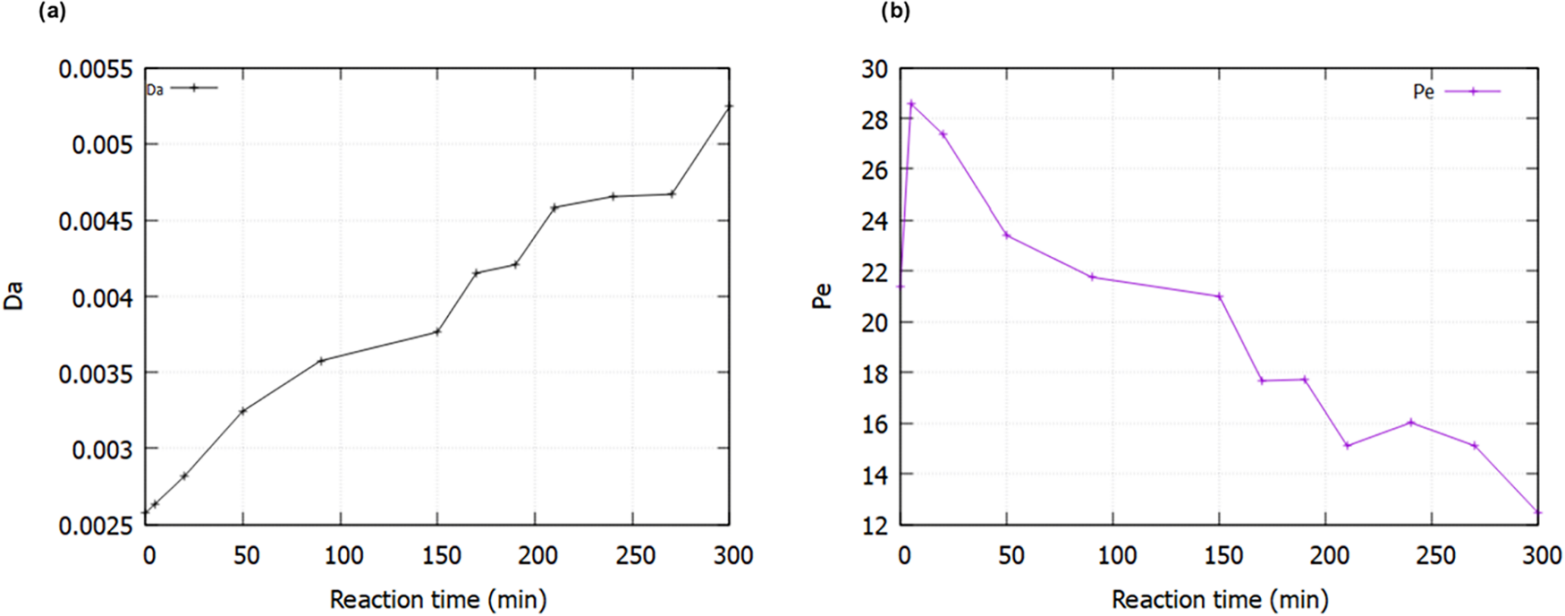
(a). Damköhler (*Da*) and (b) Péclet (*Pe*). Equations 1 and 2 as function of time.

The *PeDa* number, defined as the product of the Péclet number (*Pe*) and the Damköhler number (*Da*), characterizes the balance between advective transport and chemical reaction rates.^41^ In this study, the *PeDa* values, ranges from approximately 5.51 × 10^–2^ to 7.91 × 10^–2^, indicate a relatively low combined influence of both advection-driven transport and reaction rate. The values suggest that neither transport nor reaction overwhelmingly dominates the system; instead, both processes operate in a kinetically constrained regime. The implication of this is that mineral dissolution and associated geochemical transformations are likely to occur in a spatially distributed and temporally progressive manner, rather than being sharply localized which is observed in the gradual near-linear increase in porosity measured in the study.

The trend of *Da* and *Pe* values corresponds with those measured in Menke et al.,^7^ and Voltolini and Ajo-Franklin,^8^ although the specific values of *Da* differ by a factor of 10 and *Pe* by a factor of 100, likely due to differences in experimental conditions. For example, the flow rate used in this study (0.04 mL/min) is significantly lower than the 0.1 to 0.5 mL/min flow rates adopted in other studies,^8,15,41^ leading to reduced advection and consequently lower Péclet numbers compared to the much higher values (167–2100) observed in these studies.^8,15,41^ Also, when calculating the number of moles in calcite, the total porosity value used in the calculation was exclusively derived from the CT images, which does not account for micropores not detected by the imaging system which would also contribute to the total porosity of the sample. Supporting this, Sang and Liu^47^ showed that sc-CO_2_-acidified brine can enhance nano porosity and cause matrix erosion in tight limestones, as evidenced by combined synchrotron CT and small-angle neutron scattering. Given the higher permeability of our sample and the observed progression of dissolution over time, it is plausible that similar subresolution porosity changes occurred in our experiment but could not be captured due to limitations in imaging resolution.

### 3.5 Multiphase Simulation

One of the working hypotheses of the reactive transport process as it pertains to CO_2_ storage is that the pore structure changes due to localized dissolution, as discussed in the previous sections, which could potentially affect the residual trapping of CO_2_ in the reservoir. This trapping can be assessed by predicting capillary pressure and relative permeabilities from the network model,^48–51^ which quantify the flow and retention of CO_2_.

Relative permeabilities and capillary pressure were predicted for multiphase flow: primary drainage (CO_2_ injection in its own phase, assuming no reaction) and imbibition (subsequent reinvasion by water, again without reaction). In these simulations, the initial contact angle was set at 0° for the primary drainage cycle, reflecting strongly water-wet conditions during CO_2_ injection, while a contact angle between 80 and 100° was used for water injection, following values expected for CO_2_-brine-rock systems published in Yang et al.^52^ Capillary pressure and relative permeabilities were computed on networks derived from all the images. This approach allowed for a time-dependent estimation of brine and CO_2_ saturation levels, indicating how much CO_2_ could be immobilized in pore spaces as capillary conditions evolved over time. These simulations provide insights into the dynamic nature of CO_2_ storage potential under varying reactive transport conditions, helping to predict long-term sequestration stability in geological reservoirs.

### 3.6 Evolution of Capillary Pressure and Residual Saturation

Figure 11 presents the evolution of capillary pressure plotted against water saturation (*S*_w_) for the different images, indicating how different degrees of dissolution impact multiphase flow. From Figure 10, the entry capillary pressure (*P*_c_^entry^)—the pressure at which CO_2_ first enters the pore space is 864 Pa. As time progresses, this entry pressure gradually declines to 569 Pa after 300 min, indicating a gradual increase in the size of the throats. In addition, the maximum CO_2_ saturation increases with dissolution, as more of the pore space becomes accessible for displacement.

**Figure 10:**
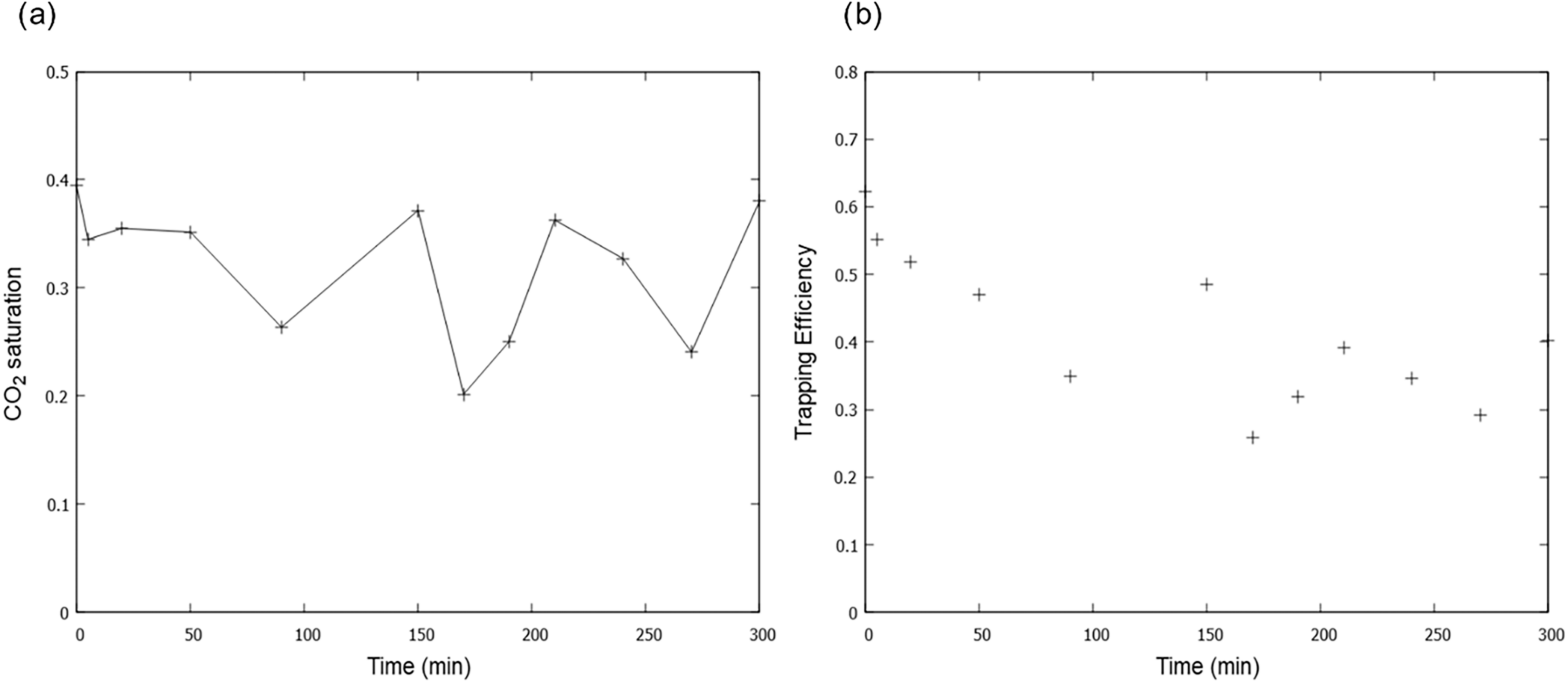
(a) Residual CO_2_ saturation after imbibition predicted from pore-space images taken at the times shown. (b) Trapping efficiency.

As well as lower capillary pressures, as the pore space becomes larger and better connected, we also show the residual saturation at the end of imbibition in Figure 11. There is no clear trend with the saturation both increasing (as now there are larger pores in which the CO_2_ can be trapped) and decreasing, as the CO_2_ is better connected, so it can escape more readily. Bachu^53^ defined trapping efficiency as the ratio of the residual CO_2_ saturation after imbibition, *S*_CO_2__^irr^, to the maximum CO_2_ saturation obtained at the end of the drainage cycle, *S*_CO_2__^max^.

3 $$\mathrm{trapping}\;\mathrm{efficiency}=\frac{S_{{\mathrm{CO}}_{2}}^{\mathrm{irr}}}{S_{{\mathrm{CO}}_{2}}^{\max}}$$

This is also plotted in Figure 11. Trapping efficiency appeared to have an initial downward trend with dissolution, as the maximum saturation reached increased.

**Figure 11:**
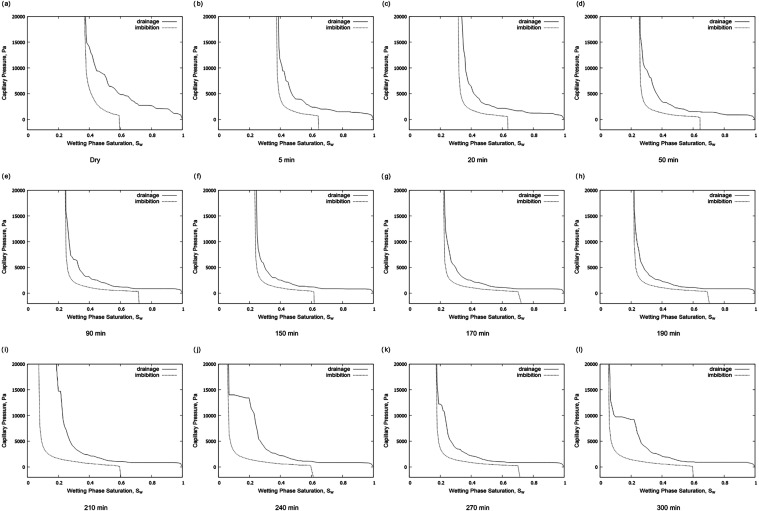
Predicted capillary pressures during two phase flow simulation: drainage in solid lines and imbibition in dashed lines (a–l).

### 3.7 Relative Permeability

Figure 12 shows the predicted relative permeabilities after drainage and imbibition simulations for the different reaction times. It can be observed that during drainage, the relative permeability of CO_2_ (*k*_rg_) remains high initially, with minimal changes up to around 90 min, indicating consistent gas flow behavior. However, as reaction time extends beyond 150 min, a noticeable decrease in *k*_rg_ at lower wetting phase saturations becomes evident. This trend suggests modifications in pore structure and connectivity consistent with the channelling effect discussed in previous sections.

**Figure 12:**
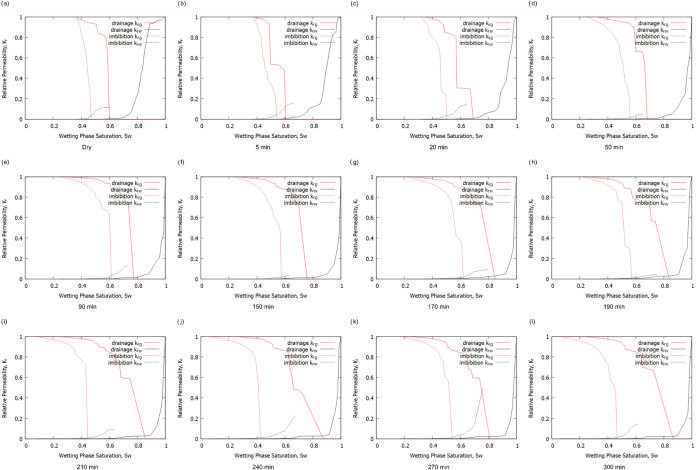
Predictions of relative permeability after drainage and imbibition simulation at each time step (a–l).

Similarly, during imbibition, the relative permeabilities display a response to reaction time, albeit more significant. Initially, the relative permeability of brine (*k*_rw_) starts at low values, highlighting the poor connectivity of the wetting phase until a high saturation is reached. As time progresses, the *k*_rw_ curves shift upward with more dissolution as the pore space becomes more accessible.

### 3.8 Implication to Field Scale

The pore-scale observations presented in this study offer valuable insights into potential mechanisms that may influence CO_2_ mobility and trapping at the field scale, particularly in far-field regions. Although the experiment was conducted under controlled laboratory conditions, the dynamic increase in permeability has implications for how CO_2_ may behave over longer time scales and larger spatial domains in subsurface storage formations.

In far-field regions, flow conditions are typically slower and more diffusion-limited compared to the near-wellbore zone.^54^ However, as demonstrated by our imaging and permeability analysis, reactive transport can enhance pore connectivity and generate preferential flow paths. When extrapolated to these regions in the reservoir, often considered passive in terms of CO_2_ migration, this suggests they could become increasingly conductive over time due to mineral dissolution, particularly along connected flow pathways.

Field evidence from projects such as Sleipner supports this idea. Time-lapse seismic monitoring at Sleipner has shown that CO_2_ plume migration is strongly influenced by heterogeneity and the presence of high-permeability fairways, rather than by uniform expansion.^55^ Although Sleipner is hosted in a siliciclastic system, the broader mechanism of flow guidance by permeability contrasts is applicable across different lithologies.^56^ In carbonates, such as the Indiana Limestone examined here, the feedback between dissolution and permeability enhancement could result in similarly preferential flow behavior, potentially allowing CO_2_ to migrate farther into the formation than would be expected under static conditions.

This evolving permeability may reduce capillary and residual trapping efficiency in zones where CO_2_ bypasses less permeable regions that would otherwise retain it. Furthermore, even modest increases in permeability can alter the pressure distribution within the reservoir.^57^ This may indirectly influence wellbore integrity and the stress regime acting on the caprock. These considerations emphasize the importance of incorporating time-dependent permeability changes and reactive transport feedback into reservoir-scale models when evaluating the long-term performance and efficiency of CO_2_ storage.

### 3.9 Limitations and Further Studies

This study provides valuable insights into the pore-scale evolution of carbonate rocks under CO_2_-saturated brine flow, utilizing high-resolution 4D synchrotron imaging. However, several limitations, many of which are inherent to synchrotron-based experimental workflows, should be acknowledged to contextualize the findings and inform future research. One important consideration is the resolution limit of the imaging system (1.625 μm), which restricts the detection of submicron-scale features, particularly microporosity and nanopores, standard in tight limestones.^47,58^ These unresolved pores may significantly contribute to the overall transport properties of the rock.^58,59^ While porosity estimates derived from segmentation accurately reflect the resolvable pore space, they likely underestimate total porosity, especially in regions affected by matrix erosion or fine-scale dissolution.^60,61^ Furthermore, permeability was inferred from image-based network modeling rather than direct pressure measurements. Direct permeability measurements were not feasible due to technical constraints related to pressure transducer stability and synchrotron timing limitations. Nevertheless, the use of image-based modeling still provided valuable insights into permeability evolution trends.

Future studies should address these limitations by adopting multiscale imaging workflows (e.g., combining synchrotron CT with nano-CT or SEM), integrating direct pressure measurements alongside pore-scale simulations, and using larger or composite samples that better capture structural/mineralogical heterogeneity. Importantly, there is a need for field-scale studies that incorporate the evolving permeability and porosity trends observed here into larger-scale reservoir models.

Bridging the scale gap between pore-scale mechanisms and reservoir-scale behavior remains a key challenge in CO_2_ storage research.

## 4 Conclusions

This study provides valuable insights into the dynamic reactive transport processes of CO_2_ in carbonate rock formations using high-resolution 4D synchrotron imaging. The observed preferential dissolution patterns, enhancement of pore connectivity, and increased porosity highlight the impact of reactive CO_2_-brine interactions on the rock’s microstructure. Key findings demonstrate that the channelling effects and resulting pore-structure changes significantly influence permeability and capillary trapping dynamics, with implications for CO_2_ sequestration efficiency. Dimensionless analyses, including Damköhler, *Da* and Péclet, *Pe* numbers, reveal a transport-dominated regime where advection facilitates reactant distribution while the reaction remains slow relative to flow rates. Importantly, these pore-scale processes have broader implications at the reservoir scale. The dynamic increase in permeability observed in this study suggests that previously flow-resisted regions could become more conductive over time due to mineral dissolution. This evolving permeability may promote preferential flow pathways, reducing the effectiveness of capillary and residual trapping, and altering reservoir pressure distribution. Such changes could influence plume migration patterns, wellbore integrity, and caprock stability over long time scales. These findings align with existing literature, providing robust validation under dynamic conditions. The integration of multiphase simulations further elucidates the temporal evolution of capillary pressure and trapping efficiency, underscoring the complex interplay between dissolution, fluid dynamics, and pore network topology. Overall, this research advances our understanding of reactive transport phenomena and offers practical implications for optimizing CO_2_ storage strategies in carbonate reservoirs.
